# Exergames and Telerehabilitation on Smartphones to Improve Balance in Stroke Patients

**DOI:** 10.3390/brainsci10110773

**Published:** 2020-10-23

**Authors:** Pablo I. Burgos, Oriana Lara, Alejandro Lavado, Ignacia Rojas-Sepúlveda, Carolina Delgado, Eusebio Bravo, Cristian Kamisato, Julio Torres, Victor Castañeda, Mauricio Cerda

**Affiliations:** 1 Department of Physical Therapy, Faculty of Medicine, Universidad de Chile, Santiago 8380453, Chile; pburgos@uchile.cl (P.I.B.); oriana.lara@ug.uchile.cl (O.L.); ignaciarojas@uchile.cl (I.R.-S.); jrtorres@uchile.cl (J.T.); 2 Department of Neuroscience, Faculty of Medicine, Universidad de Chile, Santiago 8380453, Chile; cdelgado@uchile.cl; 3 Biomedical Neuroscience Institute, Independencia 1027, Santiago 8380453, Chile; alejandro.lvd@gmail.com; 4 Integrative Biology Program, Institute of Biomedical Sciences, Faculty of Medicine, Universidad de Chile, Santiago PO Box 70031, Chile; 5 Department of Neurology and Neurosurgery, Hospital Clínico de la Universidad de Chile, Santiago 8380453, Chile; 6 Geriatrics Acute Units, Physical Medicine and Rehabilitation Service, Hospital Clínico de la Universidad de Chile, Santiago 8380000, Chile; ebravo@hcuch.cl (E.B.); ckamisato@gmail.com (C.K.); 7 Service of Physical Medicine and Rehabilitation, Clínica Dávila, Santiago 8431657, Chile; 8 Department of Medical Technology, Faculty of Medicine, Universidad de Chile, Santiago 8380453, Chile; vcastane@uchile.cl; 9 Center for Medical Informatics and Telemedicine, Faculty of Medicine, Universidad de Chile, Santiago 8380453, Chile

**Keywords:** stroke, telemedicine, neurological rehabilitation, postural balance

## Abstract

Stroke is currently the world’s second cause of disability. It can cause deficits such as postural control, and telerehabilitation could improve the therapeutic dose as well as functional results. The aim of this work is to determine the effectiveness and usability of a low-cost telerehabilitation system in patients with stroke. We developed a telerehabilitation system based on exergames on smartphones, inertial sensors, and a cloud database. We trained the balance of six participants (three men and three women) in early subacute stroke (seven weeks of progress). In addition to their conventional treatment, these participants trained for a total of nine sessions of 30 min per week, for four weeks. The telerehabilitation group was compared with a control group of four clinically similar participants (three men and one woman). Clinical and usability measurements were made before and after the training. The results show a significant improvement of 11.3 ± 3.5 points in the Berg Balance Scale, 8.3 ± 3.01 points in the Mini-BESTest, and 17.5 ± 9.87 points in the Barthel scale for the telerehabilitation group. However, only the improvements of Berg and Barthel scales were statistically higher for the telerehabilitation group compared to the control group. The proposed system achieved excellent usability on the System Usability Scale (87.5 ± 11.61). Our results demonstrate that a complementary low-cost telemedicine approach is feasible, and that it can significantly improve the balance of stroke patients; therefore, the proposed clinical strategy could potentially improve dosage and overall treatment effectiveness.

## 1. Introduction

Worldwide, stroke is the second largest contributor of disability-adjusted life years (DALYs) in developing countries, and the third largest in developed countries [1]. Postural control is among the most common alterations producing fall risk during mobility tasks, which severely affects a person’s independence and life quality [2].

Balance training studies are mostly performed in the late subacute stage or in the chronic stage [3], but recent consensus [4] in the stroke rehabilitation literature suggests the need for studies on the acute and/or early subacute phase to demonstrate that training in these stages where there is a major level of plasticity can lead to better results than those of spontaneous recovery [4]. Additionally, there is a natural resistance to remote asynchronous balance training because of fall risks, which has encouraged health systems to decrease the chance of falls particularly in elderly [5,6].

Telerehabilitation studies have reported that the use of telemedicine using exergames and sensors (custom, Inertial Measurement Units or IMUs, videocameras) to estimate posture has improved patients’ coverage, adherence, and clinical results [7,8]. Simple video conference or telephone systems, or recorded videos for self-education strategies have increased patients’ exercise rehabilitation dose (frequency and intensity) and balance results, which may be similar or even better than those of conventional therapy [8,9]. Although simple systems increase therapist time (synchronous systems), complex systems seem extremely difficult to use outside the research protocols [10,11,12] or in a mobile manner (with an easy transition from hospitals, labs, and homes).

Thus, there are two major challenges in stroke treatment using telemedicine tools: (1) to provide evidence of the effectiveness and safety of acute or early subacute remote balance training, and (2) to propose simple and cost-effective systems and protocols to promote a massive incorporation into clinical practice.

In this work, we propose a set of exergames for balance training by means of smartphones and IMUs. The main aim is to develop an effective complementary ambulatory treatment to improve balance in stroke patients during the early subacute phase (one–three months) in a cost-effective, safe, and simple manner.

## 2. Materials and Methods

### 2.1. Participants

Stroke patients with 6 to 8 weeks after the onset of lesion were invited to participate. All patients were undergoing physical therapy at the Hospital Clínico de la Universidad de Chile (HCUCH) or at Hospital San José (HSJ), both located in Santiago, Chile. Patients were invited to participate between August 2018 and July 2019 after approval of the HCUCH ethical committee (Protocol number 923/17), in agreement with the Declaration of Helsinki, and individual signing of the informed consent. Participants’ demographics, stroke details, comorbidities, and medications are shown in Table 1. Participants’ inclusion criteria were to have a biped time > 30 s, a Berg Balance Scale (BBS) < 50, and at least one caregiver at home.

**Figure 1:**
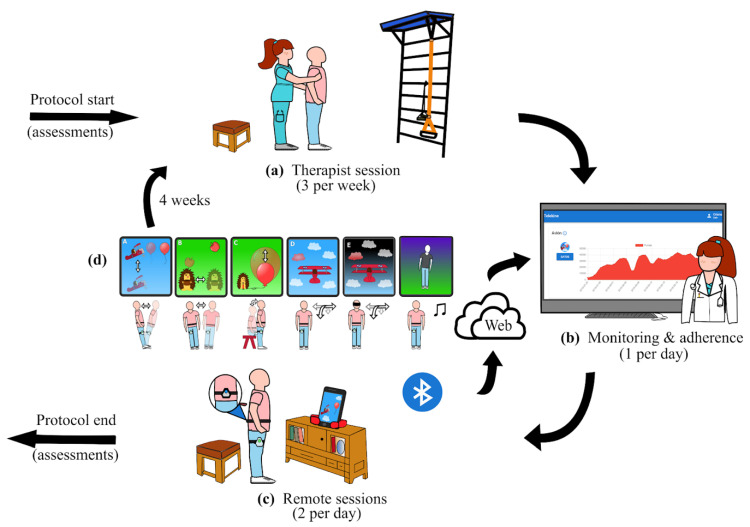
The proposed protocol: (**a**) The protocol started with a balance assessment and a therapist session at the hospital’s physical therapy unit, followed by rehabilitation sessions 3 times per week; (**b**) after equipment delivery as well as participant and caregiver training, the therapist remotely monitored home progress using the web platform; (**c**) the participant exercised at home using the proposed system while games scores and IMU recordings were sent to the platform database for monitoring; (**d**) after 4 weeks, the protocol ended by repeating the balance assessment.

**Figure 2:**
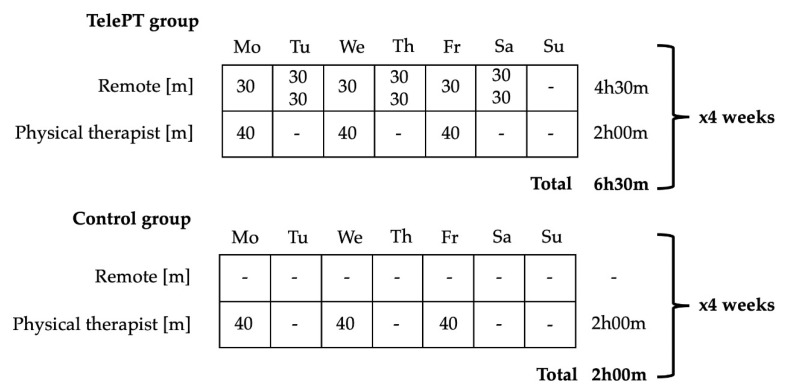
Protocol dose and session distribution during an example week.

Volunteer stroke participants were randomly assigned to two groups: Control and TelePT. Both groups received their standard rehabilitation treatment at the hospital site, as illustrated in Figure 1a and Figure 2 (3 sessions of 40 min per week of physical therapy for 4 weeks). In addition, the TelePT group received 9 sessions of 30 min per week for 4 weeks (see weekly distribution in Figure 2). In each session, participants trained in balance tasks using smartphone-based exergames controlled by body motions, as illustrated in Figure 1c.

### 2.2. Caregivers

When the equipment was delivered at home, both the participant and caregiver were instructed on safety recommendations and how to use the equipment (if a second caregiver was available, they also received the same training). Safety guidelines included locating a safe place to exercise and learning how to assist the participants when required. The training about the use of equipment includes sensors’ placement and connection, calibration stage, starting games, and the participation in an exergame of reactive balance (see Video S2).

### 2.3. Assessments

Balance was assessed using BBS and Mini-BESTest (MBT), and functional independence was assessed using the Barthel Index (BI). All assessments were applied at protocol start and end (Figure 1). Telemedicine treatment was carried out by a trained physical therapist. In addition, the System Usability Scale (SUS) [13] was used to measure user experience. The SUS consists of 10 questions about frequency of use, complexity, difficulties using the system, security, and user’s previous knowledge. SUS scores range from 0 to 100 points, with 70–80 rated as “good”, 80–90 as “excellent”, and 90–100 as “best imaginable” [13].

### 2.4. Telemedicine Equipment

An Android-based smartphone (Samsung Galaxy J7 Prime 2016) integrated with two wireless inertial movement sensors (IMUs) [14] was used. Sensors were positioned using velcro fasteners at the lumbar level (posterior middle line) and at the anterior thigh of the paretic side. Participants interacted with our custom-developed Android app, including a calibration stage that recorded participants’ stability limits (see Video S2). The physical therapist, on the other hand, operated a customized web platform to monitor participants’ activity and adherence (see Video S3).

### 2.5. Exergames

Participants were able to modify their own routine difficulty level as they progressed in the protocol, which was determined by their calibration results. Six exergames were specifically built in our app to specifically promote the following: (1) antero-posterior increase in stability limits, (2) medio-lateral increase in stability limits, (3) anticipatory adjustments in sit to stand transfer, (4) standing postural oscillations reduction, (5) reactive balance, and (6) training of dynamic anticipatory postural control through dancing. A detailed description of exergames with rehabilitation goals, feedback type, games targets, used sensors, and difficulty progression is shown in Table S1 and examples are illustrated in Figure 1d and in Videos S1 and S2.

### 2.6. Clinical Protocol

The equipment was delivered to each study participant at home, with alarms set up 15 min ahead of exercise times. If a participant did not perform the session, the therapist called the participant via telephone. Therapist monitoring was done by connecting to the web platform and watching games scores daily at the scheduled session time or afterwards based on therapist availability (see Video S3). The main idea of the proposed participant/physical therapist interaction flow is to keep standard interaction and to increase exercise dose by using the developed platform (as shown in Figure 1). Therefore, a physical therapist was in charge of equipment delivery, initial training, games prescription according to participant characteristics, safety precautions, and daily participant contact via WhatsApp’s phone app. Daily contact was kept as a way to increase protocol adherence and to be responsive to any detected technical problem promptly.

### 2.7. Statistics

A statistical analysis was carried out using R [15]. The comparison between TelePT and Control groups was computed using the 2-way ANOVA test with repeated measures, using intervention type (TelePT/Control) and session (PRE/POST) as factors and the R “aov” function. Using the BBS pre-score, we removed one control participant (BBS score 20) from another clinical category [16] and also identified them mathematically as an outlier. Cohen’s d effect size was calculated between PRE and POST treatment scores to obtain the study’s results and to compare them with those of other studies in the Discussion section. When several references were available, the one with the highest effect size was selected for comparison.

## 3. Results

### 3.1. Telerehabilitation Training Effects

**Figure 3:**
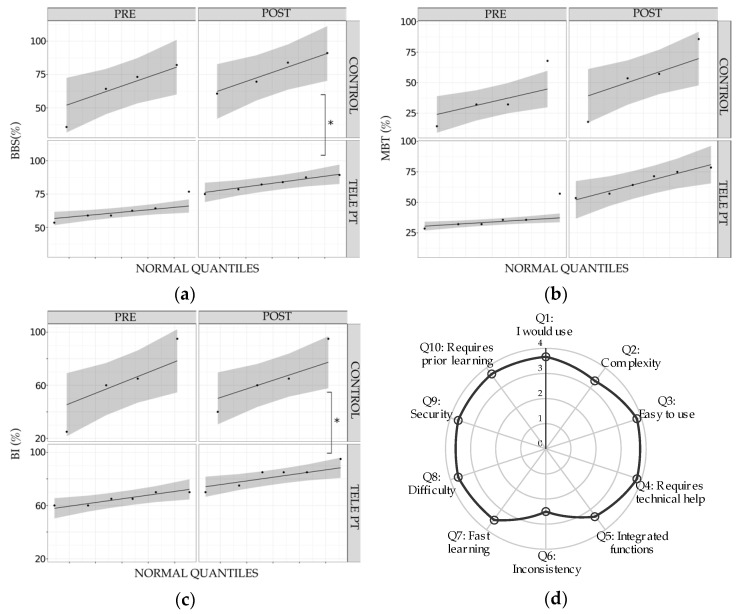
Balance and functional progress in telerehabilitation group, and system usability: (**a**–**c**) improvement within PRE and POST sessions by group (TELEPT vs. CONTROL): BBS: Berg Balance Scale, MBT: MiniBesTest, and BI: Barthel Index. The points represent the individual scores in a qq plot, the gray area represents the dispersion (theoretical confidence interval at 95%), and the asterisk shows significant differences of PRE–POST variation between groups (ANOVA 2-WAY RM); (**d**) usability evaluation with SUS scale (10 questions), from 0 to 4.

PRE and POST training clinical scales were registered for each study participant, as shown in Table 1. Clinical scores are also presented as percentages with respect to the maximum value on each scale in Figure 3a–c. Balance results improved in the BBS, with mean values of PRE = 35 ± 4.42 (62.50% ± 7.91), POST = 46.33 ± 3.01 (82.67% ± 5.37), and MBT PRE = 10.33 ± 2.87 (36.89% ± 10.26), and POST = 18.67 ± 2.81 (66.67% ± 10.01) with a statistically significant variation within PRE and POST (F(1/5) = 60.84, *p* < 0.001 and F(1/5) = 45.96, *p* = 0.001, respectively). Functional independence, measured by BI, also improved in the study group with PRE = 65.00 ± 4.47, and POST = 82.50 ± 8.80. There was also a statistically significant variation within PRE and POST times (F(1/5) = 18.85, *p* = 0.007). Percentage comparisons are shown in Figure 3a.

### 3.2. Comparison with Conventional Treatment Results

The data of control participants of PRE and POST training clinical scales are also shown in Table 1. In comparison with the control participants, BBS variation PRE–POST for the study group was higher, with 20.20% ± 6.36 vs. 12.50% ± 8.63, with a statistically significant difference of the variation between groups (F(1/7) = 9.15, *p* = 0.019; Cohen-d = 2.98). For MBT PRE–POST variation, it was 29.7% ± 10.75 in the telerehabilitation group and 16.96% ± 9.39 in the control group, without significant differences between groups (F(1/7) = 1.61, *p* = 0.245; Cohen-d = 2.94). Functional independence (BI) in participants trained with our telerehabilitation system was higher compared to the controls: 17.50 ± 9.87 vs. 3.75 ± 8.53, with a significant difference between groups (F(1/7) = 7.97, *p* = 0.025; Cohen-d = 2.50).

### 3.3. User Experience

The average SUS score was higher than 80 (87.5 ± 11.61), which can be interpreted as an excellent system user usability level [13]. In Figure 3d, average SUS scores for each of the 10 questions are shown. Most items score higher than 3 in a Likert scale from 0 to 4. Only consistency (P6) was evaluated lower than 3, which may be interpreted thanks to open comments by participants: two reported difficulties in performing initial calibration, one suggested increasing the screen size, and another suggested increasing the games difficulty, while three participants suggested an increase in resting time between game repetitions, especially for the sit to stand transfer game.

## 4. Discussion

This study reports changes of balance, functional independence, and usability in participants with stroke in the subacute stage who use telerehabilitation complementary to conventional rehabilitation. The results were statistically significant for the within effect in the telerehabilitation group for BBS, MBT, and the BI scales; however, these results were only significant in the between comparisons for BBS and BI.

In comparison with the literature on balance, BBS effect size for the study group was higher in our study group, with 2.98 vs. 1.82, respectively [13,18]. The compared study had these features: it was an RCT with 24 stroke participants; the onset of training was approximately at week 12 after stroke (late subacute stage); the training lasted four weeks, with five sessions of 30 min per week, and it used the space balance 3D training system (CyberMedic Co.) in a health center plus 1 h of conventional therapy per day. Thus, in comparison to our study, it consisted of 7.5 h per week of exercise in a high-end equipment vs. our proposed 6.5 h per week strategy without an adherence report.

Our study’s MBT effect size was 2.94 in the TelePT group, which contrasts with 0.43 reported in a conventional rehabilitation study [19]. That study’s methodology was a prospective observational design with 26 stroke participants in the subacute stage (without weeks details in the reference) measured before and after 10 one-hour sessions over two weeks of conventional therapy focused on balance training without an adherence report.

Functional independence (BI) in participants trained with our telerehabilitation system was slightly higher compared with the expected literature effect, with 2.50 vs. 1.95, respectively [20]. The study used for the comparison was an RCT with 190 stroke participants after two weeks post-stroke (early subacute phase). The intervention was home visits and telephone calls by a nurse to coordinate the participants with the rehabilitation services compared to the conventional interaction without an adherence report.

Study group improvements of BBS (11.33 points), MBT (18.67 points), and BI (17.5 points) are also higher than reported minimal detectable changes (MDC) for BBS (6.9 [21]), MBT (3.5 points [19]), and BI (16.2 points [22]). Thus, our results are in line with the latest systematic reviews, which report similar or better improvements of impairments and functional independence in telerehabilitation compared to conventional therapy [7,8,9].

In other telerehabilitation protocols, the best result in BBS has been an effect size of 1.29 [10,12]. We did not find any comparable results using MBT. Regarding functional independence, the best BI variation in other telerehabilitation protocols shows an effect size of 1.5 [23]. Consequently, after comparing with existing literature, our protocol shows better results in balance and independence outcomes, which could be explained by the onset and intensity of our proposed training [10,11,12].

As for usability (SUS), the system had an excellent acceptance among participants. This result may be due to the constant interaction by means of a smartphone app, the use of personalized games instead of standardized games, [10] and the chance to provide performance feedback.

In terms of costs, the proposed system initial costs were as follows: a smartphone (USD 200 if new, though the participant’s smartphone may be used), two IMU sensors with a charger (USD 200), mobile internet (USD 10), servers, and engineer time. We also estimate a worst case of 1 h per week (1 min per day when everything was fine, and 10 min when something fails, for six days) of physical therapist work in remote monitoring (4 h for the entire protocol, or USD 100 in Chile). This extra physiotherapist hour allows an additional 4 h 30 min hours of a patient’s effective training per week. However, at least routine remote monitoring could be performed by a non-specialist.

In spite of its positive results, the main limitation of this study is the small number of participants who underwent training due to the study’s inclusion criteria. Similarly, even though in our proposed system we mainly focus on dose (increasing exercise) and plasticity (choosing early subacute stage) factors, we cannot discard the spontaneous recovery contribution (residual structure and physical activity after stroke), as pointed out by Krakauer et al. [24]. Further, TelePT-Control groups differences could be explained by the intervention itself or the increase in contact hours.

In relation to the technological aspects, adherence data sending, sensors connection stability, and calibration stability are areas that need further improvement. Lastly, if massive deployments were to be implemented, remote smartphone control for support will be needed.

## 5. Conclusions

Our pilot balance remote training system improves postural control significantly more than other studies of conventional or remote rehabilitation in stroke patients. The latter may probably result from the higher dosage of our training and the chosen early subacute stage. Furthermore, the protocol was simple and safe for participants and caregivers (without adverse reports). Finally, the clinical and usability results confirm the feasibility of using a smartphone with low-cost sensors as a tool that can democratize access and allow personalization based on the needs of each patient.

## Figures and Tables

**Table 1:** Participants’ description. Summary of participants description and detailed scale values. S: sex, A: age in years, R: right, L: left, I: ischemic, H: hemorrhagic, TACS: total anterior circulation stroke, PACS: partial anterior circulation stroke, POCS: posterior circulation syndrome, LACS: lacunar stroke.

ID	S	A	Diagnostic ^1^	Comorbidities	Drugs	BBS ^2^	MBT ^2^	BI ^2^
TELE_PT 750352	F	62	POCS/R/I/8	Arterial Hypertension, Diabetes Mellitus type 2, High cholesterol	Losartan, Metformin, Atorvastatin, Aspirin	33/44	9/15	60/95
TELE_PT 981,283	M	46	PACS/R/H/7	Arterial Hypertension, Diabetes Mellitus type 2, Smoking, Dyslipidemia	Metformin, Atorvastatin	35/47	9/20	65/85
TELE_PT 798,056	F	62	TACS/L/H/6	Gastric Bypass (2015)	Sertraline	30/42	8/18	60/75
TELE_PT 361,739	M	63	PACS/L/I/7	Arterial Hypertension	Losartan	33/50	10/22	70/85
TELE_PT 194,267	F	54	LACI/L/I/8	Arterial Hypertension, Diabetes Mellitus type 2, Dyslipidemia	Losartan, Metformin, Insulin, Sertraline, Aspirin	36/46	10/16	70/85
TELE_PT 210,586	M	55	POCS/L/I/7	Arterial Hypertension, Diabetes Mellitus type 2	Aspirin, Losartan, Metformin	43/49	16/21	65/70
CONTROL 955,948	M	62	PACS/L/I/7	Dyslipidemia	Aspirin, Atorvastatin	46/51	19/24	95/95
CONTROL 328,977	M	79	LACS/R/I/7	Dyslipidemia, Arterial Hypertension	Atorvastatin, Carvedilol	41/47	9/15	60/65
CONTROL 254,683	M	61	PACS/R/I/6	No	Aspirin	20/34	4/5	25/40
CONTROL 254,688	F	69	PACS/R/I/6	Dyslipidemia, Arterial Hypertension	Losartan	36/39	9/16	65/60
TELE PT PRE/POST Average						35.00/46.33	10.33/18.67	65.00/ 82.50
CONTROL PRE/POST Average						35.75/42.75	10.25/ 15.00	61.25/ 65.00

^1^ Bamborf [17]/side/etiology/weeks after stroke onset; ^2^ PRE/POST.

## Supplementary Materials

The following are available online at https://www.mdpi.com/2076-3425/10/11/773/s1, Video S1: Demo session, and a participant training session, Video S2: Calibration stage and games demo, Video S3: Website for therapist monitoring, Table S1: Games’ detailed description.
