# Supplementary material for: Exergames and Telerehabilitation on Smartphones to Improve Balance in Stroke Patients
Source: Brain Sci. 2020 Oct 23;10(11):773. doi: 10.3390/brainsci10110773 (PMC7690853; doi:10.3390/brainsci10110773)
Supplement: Supplementary file 1 [file brainsci-10-00773-s001.zip › SupplementaryFiles/SupplementaryTable 1.pdf]

Supplementary Table 1. Games detailed description.

Exergame	Rehabilitation goal	Feedback	Game Target	Sensors	Difficulty
"Airplane" 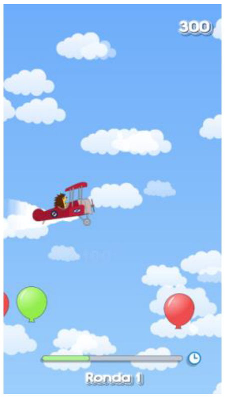	Antero-posterior increase of stability limits.	Visual Airplane controlled by the anterior-posterior body inclination.	Pop balloons with the movements of airplanes.	Lumbar sensor getting the inclination of body.<br><br>Thigh Sensor detect hip strategy sending a warning to promote the ankle strategy.	Stable during the session. Could increase depending on calibration stage (recording of current stability limits).
"Fruits" 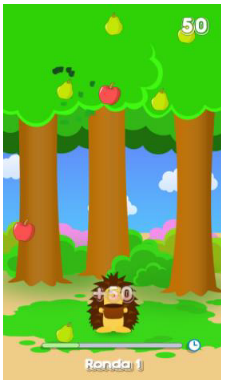	Medio-lateral increase of stability limits	Visual Hedgehog controlled by medio-lateral body inclination.	Catch falling fruits moving the hedgehog.	Lumbar sensor getting the inclination of body	Stable during the session Could increase depending of calibration stage (recording of current stability limits).
"Day clouds" 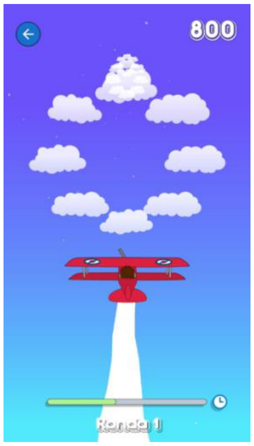	Standing postural oscillations reduction.	Visual Airplane moves by medio-lateral or anterior-posterior body inclination.	Advance through the sky without touching clouds.	Lumbar sensors detecting the static position.	Stable during the sessions.

“Night clouds” 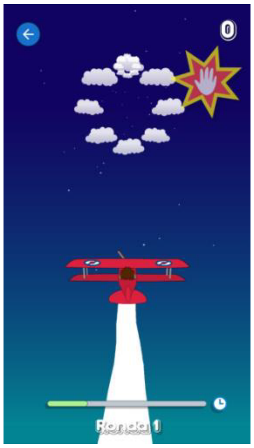	Reactive balance standing with eyes closed.	As “Day clouds” and participants try to maintain the posture while the caregiver does a small push to the participant.	Advance through the sky without touching clouds.	Lumbar sensors detecting the static position.	Stable during the sessions.
“Dance” 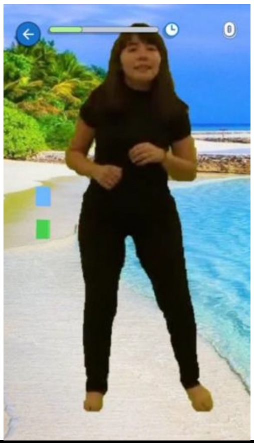	Training of dynamic anticipatory postural control through dancing.	Game score, indicating hits and errors of performance.	Participants had to imitate the movements of the dance model presented in the game.	Lumbar sensor and a sensor located in the thigh.	Stable during the sessions.
